# The Effect of Family Support on Self-Management Behavior in Postoperative Cardiac Surgery Patients: A Cross-Sectional Study

**DOI:** 10.31083/RCM31261

**Published:** 2025-05-20

**Authors:** Ting Shen, Qiuhong Chen, Ting Leng, Mengling Gu, Lin Luo, Furong Jiang, Xiahong Huang

**Affiliations:** ^1^Division of Critical Care Medicine, Deyang People’s Hospital, 618000 Deyang, Sichuan, China

**Keywords:** patients after cardiac surgery, cardiac rehabilitation, family support, self-management, correlation analysis

## Abstract

**Background:**

Cardiac rehabilitation (CR) serves as a critical component in ongoing care for cardiovascular disease patients, improving postoperative anxiety and depression in cardiac surgery patients while reducing readmission rates and mortality. However, patient completion rates for CR programs remain low due to insufficient awareness and lack of social support. This study aimed to investigate the impact of family support levels on self-management behaviors in postoperative cardiac surgery patients, providing a basis for family-based cardiac rehabilitation interventions.

**Methods:**

This cross-sectional survey involved 76 patients who had undergone major vascular surgeries one month prior and were subsequently discharged from the hospital’s cardiology department. Participants completed questionnaires assessing demographic details, family support, psychological status, and self-management practices. Logistic regression analysis identified factors influencing perceived social support from family (PSS-Fa), while correlation analyses examined relationships between family support and self-management behaviors.

**Results:**

The mean PSS-Fa score was 10.82 ± 1.50, and the average self-management behavior score was 140.80 ± 20.46. Female gender, marital status, and educational attainment significantly influenced higher family support scores (*p* < 0.05). For the univariate analysis, key determinants of better self-management included age, educational level, marital status, household income, type of medical insurance, presence of comorbidities, cardiac function classification, and psychological states indicative of anxiety or depression (all *p* < 0.05). Multiple linear regression analysis showed that PSS-Fa, age, and education level significantly influenced self-management behaviors in postoperative cardiac patients. Family support and education level had a positive effect, while age had a negative impact. The model’s overall fit statistics are *R^2^* = 0.821 and *F* = 33.722 (*p* < 0.05). Pearson’s correlation analysis revealed a positive association between family support and overall self-management behaviors (*r* = 0.303, *p* < 0.05), particularly in nutrition management, exercise adherence, self-monitoring, and timely medical consultations.

**Conclusion:**

This suggests that the role of family support should be fully considered in developing CR programs in the future, and targeted interventions should be implemented to enhance this support, thereby potentially improving patient outcomes and adherence to CR programs.

## 1. Introduction

Cardiac rehabilitation (CR) is a comprehensive outpatient program that combines exercise training and educational interventions to promote lifestyle changes, control risk factors, and implement secondary prevention strategies. Studies have shown that CR is not only effective in alleviating anxiety and depressive symptoms but also significantly improves patients’ quality of life and reduces readmission and mortality rates in patients after cardiac surgery [1, 2]. However, despite these substantial benefits, many eligible individuals fail to fully engage in CR programs as intended. Indeed, participation rates for CR are notably low in China, ranging from 19% to 45% [3, 4]. Several factors contribute to this underutilization, including personal characteristics such as age, gender, and level of education, as well as socioeconomic factors such as income and social support networks [5, 6]. Furthermore, post-surgical patients frequently experience heightened emotional distress, which complicates self-management efforts and adherence to prescribed CR protocols [7, 8].

Family support is a key component in the social support system that provides patients with financial, material, and psychological assistance and significantly enhances their self-management skills [9]. Given the limited accessibility of traditional CR programs, Chinese CR experts advocate for home-based cardiac rehabilitation (HBCR) as an alternative for stable patients [10]. A study conducted by McDonagh *et al*. [11] demonstrated that HBCR was comparable to facility-based CR programs regarding clinical outcomes and health-related quality of life while validating its safety and efficacy. However, the success of HBCR hinges on patient engagement and optimal self-management practices.

Although some studies have demonstrated the positive impact of family support on the self-management behaviors of patients after cardiac surgery, most existing studies have focused on qualitative analyses, meaning the quantitative assessment of the relationship between family support and self-management remains underexplored [12, 13]. Additionally, there is insufficient exploration into how cultural differences impact the efficacy of family support, especially within the Chinese context [14, 15].

Self-management is vital to patients following cardiac surgery since it encompasses a broad spectrum of elements, including managing anticoagulation therapy, monitoring symptoms, regular follow-up assessments, preventing infection, lifestyle modification, and emotional regulation [16]. Although systematic reviews have emphasized the importance of self-management in the context of HBCR [17, 18], there remains a lack of research on the specific level of self-management and its influencing factors in these patients. Therefore, this study aimed to assess the level of family support and related self-management behaviors one month after cardiac surgery to explore the impact of family support on self-management outcomes and to provide a scientific basis for HBCR interventions.

This manuscript presents a reanalysis of data that was previously published in ‘The Effect of Family Support Level on Psychological State and Self-Management Behavior in Patients Undergoing Cardiac Surgery’ in Prevention and Treatment of Cardiovascular Diseases. It not only includes original findings but also offers deeper insights into the influence of varying levels of family support on postoperative self-management behaviors. Specifically, it provides additional perspectives on how different degrees of family support can impact patient outcomes, thereby offering more targeted guidance for interventions aimed at improving these patients’ self-management practices.

## 2. Materials and Methods

### 2.1 Design and Sample

This study employed a cross-sectional correlation design to investigate patients who underwent cardiac surgery at our hospital between January 2024 and June 2024. Inclusion criteria: (1) age ≥18 years; (2) having undergone cardiac surgery, including heart valve replacement or plasty, coronary artery bypass grafting, or congenital heart disease surgery; (3) no language communication barriers, capable of completing the survey in either written or oral form; (4) informed consent and willingness to participate in the study. Exclusion criteria: (1) presence of consciousness disorders or mental illness; (2) inability to perform physical activity due to other serious diseases; (3) history of cardiac malignant tumor surgery; (4) discharge with a heart function class IV (New York Heart Association (NYHA)); (5) concurrent participation in another research study.

This study employed power analysis to determine a suitable sample size. Based on existing literature reporting the correlation between self-management behaviors and family support in postoperative cardiac surgery patients [12], we anticipated an effect size of 0.5 (considered a medium effect) to achieve 80% power and to detect this effect size with a two-tailed significance level of α = 0.05. The power analysis indicated a minimum sample requirement of 72 participants; considering potential dropout rates and other unforeseen factors such as missing data or participant withdrawal, we decided to recruit an actual sample size of 76 participants.

The hospital’s Medical Ethics Committee (2024-04-074-K01) reviewed and approved the study protocol, and all participants provided written informed consent.

### 2.2 Procedure and Data Collection

Four trained researchers conducted a telephone questionnaire to investigate the patients who met the inclusion and exclusion criteria after cardiac surgery. The survey included demographic data such as gender, age, education level, marital status, etc. Clinical data and disease-related characteristics of patients were also obtained by reviewing medical records. Self-reported questionnaires were used to assess family support, anxiety, depression, and self-management behavior.

Before conducting the survey, each researcher explained to the participant the purpose, content, anonymity, free of charge, and necessary study precautions. Once participants fully understood these aspects, informed consent was provided. To ensure that patients could clearly understand the questions, complex terminology was avoided as much as possible during the survey to ensure that the questions were easy to understand and answer. After completing the study, researchers reviewed the data for completeness and reasonableness. For any missing or unclear responses, the researcher contacted the participant promptly to correct or add information as needed.

### 2.3 Sample Characteristics

All patients completed a general information questionnaire (including gender, age, education level, marital status, per capita monthly household income, type of medical insurance, comorbidities, cardiac function grade, etc.). Additionally, the family support self-rating scale, general hospital anxiety and depression scale, and self-management behavior scale were simultaneously measured.

#### 2.3.1 Family Support

The perceived social support from family (PSS-Fa) [19] was used to assess the level of family support each patient received. The scale contains 15 entries with a total score from 0 to 15; 0 to 10 represents a low level of family support, and ≥11 represents a high level of family support; thus, higher scores indicate a higher level of family support in patients.

#### 2.3.2 The Generalized Anxiety Disorders Scale

The General Hospital Anxiety and Depression Scale (HADS) was used to detect psychological states. The scale contains two categories of anxiety and depression, each containing seven items, and can be scored on a scale of 0–3, with total scores ranging from 0–21 points. The higher the score, the more serious the depression or anxiety. For each subscale, a score from 0 to 7 indicates no anxiety or depression, 8 to 10 indicates mild depression or anxiety, 11 to 14 indicates moderate depression or anxiety, and 15 to 21 indicates severe depression or anxiety [20].

#### 2.3.3 Self-Management Behaviors

The self-management behavior scale for heart valve replacement surgery, designed by Wang [21], was used to assess patients’ self-management ability. This scale includes six dimensions comprising medication, nutrition, health care, exercise, self-monitoring, and medical-seeking, with 38 entries. A Likert 5-point scale with a maximum score of 190 was used. The content validity of the scale was 0.937, the content validity of each dimension was 0.93–1.00, and the Cronbach’s alpha coefficient was 0.94.

### 2.4 Data Analysis

Data preprocessing: we conducted preliminary checks on all collected data to ensure quality, including cleaning outliers and consistency.

Handling of missing values: for cases with missing data, we employed multiple imputation techniques to fill in the gaps, preserving the integrity and completeness of our sample.

To summarize the sociodemographic and clinical characteristics of the sample, we conducted descriptive statistical analyses. Categorical variables are presented as frequencies and percentages (%), and the chi-square test or Fisher’s exact test was employed to compare proportions between groups. Continuous variables that met the assumptions of normality were described using the mean and standard deviation ($\bar{x}$ ± s), and independent samples *t*-tests were employed to compare mean values between the two groups. Continuous variables that did not meet the assumptions of normality were reported as medians with interquartile ranges (median (IQR)), and the non-parametric Mann–Whitney U test was used for between-group comparisons. For comparisons involving continuous variables across multiple groups, one-way ANOVA was applied if the data met the assumptions of normality and homogeneity of variances; otherwise, the non-parametric Kruskal–Wallis H test was utilized.

Given the aim of our study—to explore a simple linear relationship between levels of family support and self-management behaviors—we primarily chose to use Pearson’s correlation coefficient. Scatter plots were visually inspected to confirm the linearity assumption before applying Pearson’s correlation. This bivariate approach was deemed suitable for exploring direct associations without modeling complex interactions between multiple predictors.

Multiple linear regression analyses were conducted to identify key factors influencing self-management behaviors. Variables included in the regression model were selected based on their theoretical relevance and preliminary univariate analyses.

Statistical significance was set at a two-tailed *p*-value ≤ 0.05. All statistical analyses were performed using SPSS version 22.0 (IBM Corp., Armonk, NY, USA).

## 3. Results

### 3.1 Demographic Characteristics of the Sample

This study included 76 patients, 48 (63.15%) of whom were female and 28 (36.85%) were male, with a mean age of 52.81 years (SD = 11.96). Of the participants, 39 (51.31%) reported high levels of family support, as indicated by a mean PSS-Fa score of 10.82 (SD = 1.50), whereas 37 (48.68%) reported lower levels of family support. Table 1 shows the distribution of sociodemographic and clinical characteristics of the participants.

**Table 1.** **Comparison of family support levels among patients with different sociodemographic and clinical characteristics**.

Characteristics		N (%)	PSS-Fa	Expected N	*t*/*F*	*p*-value
Gender					–3.041	0.003*
	Male	28 (36.8)	10.2 ± 1.5	27.5		
	Female	48 (63.2)	11.2 ± 1.4	48.5		
Age					0.597	0.553
	≤45	15 (19.7)	11.2 ± 1.8	15.2		
	45–60	36 (47.4)	10.8 ± 1.4	35.6		
	>60	25 (32.9)	10.7 ± 1.5	29.2		
Education level					2.805	0.046*
	Primary education	19 (25.0)	10.1 ± 1.0	18.8		
	Junior high school	35 (46.1)	10.9 ± 1.5	35.5		
	High school	16 (21.1)	11.1 ± 1.5	16.4		
	University and above	6 (7.8)	11.8 ± 1.9	7.3		
Marital status					15.600	0.000*
	Married	53 (69.7)	11.2 ± 1.3	52.4		
	Unmarried	7 (9.2)	11.3 ± 1.7	7.1		
	Divorced/widowed	16 (21.1)	9.2 ± 0.6	16.5		
Monthly income, USD					2.992	0.056
	≤280	9 (11.8)	9.8 ± 1.4	9.6		
	280–700	47 (61.8)	10.9 ± 1.4	46.5		
	≥700	20 (26.4)	11.2 ± 1.6	20.9		
Medical insurance type					3.308	0.042*
	Self-financing	5 (6.6)	10.4 ± 1.7	5.1		
	Resident medical insurance	48 (63.1)	10.6 ± 1.4	47.5		
	Employee medical insurance	23 (30.3)	11.5 ± 1.6	22.4		
Diagnosis					1.123	0.346
	AD	6 (7.8)	11.0 ± 1.9	6.0		
	CT	8 (10.5)	11.5 ± 1.3	8.2		
	VHD	60 (78.9)	10.8 ± 1.5	60.1		
	ASD	2 (2.6)	9.5 ± 0.7	2.7		
Comorbidities					0.586	0.626
	Hypertension	20 (26.3)	10.7 ± 1.7	20.0		
	Diabetes	13 (17.1)	10.5 ± 1.3	13.3		
	Others	7 (9.2)	11.0 ± 2.0	7.2		
	None	36 (47.4)	11.0 ± 1.4	36.5		
Cardiac function					0.950	0.392
	NYHA class I	12 (15.8)	11.3 ± 1.7	12.3		
	NYHA class II	31 (40.8)	10.8 ± 1.3	30.7		
	NYHA class III	33 (43.4)	10.6 ± 1.6	32.0		
Anxiety/depression					2.066	0.134
	Low	18 (23.7)	11.0 ± 1.5	18.1		
	Medium	43 (56.6)	11.0 ± 1.4	42.6		
	Moderate to severe	15 (19.7)	10.1 ± 1.7	15.3		

Note: PSS-Fa, perceived social support from family; AD, aortic dissection; CT, cardiac tumor; VHD, valvular heart disease; ASD, atrial septal defect; NYHA, New York Heart Association. NYHA classes are defined as follows: ∙ NYHA class I: No limitation of physical activity. Ordinary physical activity does not cause undue fatigue, palpitation, or shortness of breath. ∙ NYHA class II: Slight limitation of physical activity. Comfortable at rest. Ordinary physical activity results in fatigue, palpitation, or shortness of breath. ∙ NYHA class III: Marked limitation of physical activity. Comfortable at rest, but less than ordinary activity causes symptoms. The *t*-test was used for gender comparisons; ANOVA was used for age, educational level, marital status, per capita monthly income, medical insurance type, diagnosis, complication, cardiac function, and anxiety/depression comparisons; * indicates significance: *p* ≤ 0.05.

Statistical analysis revealed no significant differences in PSS-Fa scores across various demographic characteristics such as age, monthly family income, diagnosis, presence of comorbidities, cardiac function, and levels of anxiety or depression (all *p* > 0.05). However, subgroup analyses indicated that certain groups exhibited significantly higher PSS-Fa scores compared to their counterparts (all *p* < 0.05):

∙ Gender: specifically, females exhibited significantly higher PSS-Fa scores compared to males (t (74) = –3.041, Cohen’s d = 0.72, 95% CI [0.28, 1.16]), suggesting a medium to large effect size.

∙ Education level: individuals with at least a senior high school education demonstrated significantly higher PSS-Fa scores (*F* = 2.805, *p* = 0.046*, η^2^ = 0.001).

∙ Medical insurance type: participants covered by employee medical insurance had significantly higher PSS-Fa scores (*F* = 3.308, *p* = 0.042*, η^2^ = 0.001).

∙ Marital status: married participants exhibited significantly higher PSS-Fa scores than unmarried or divorced/widowed (*F* = 15.600, *p* < 0.001, η^2^ = 0.006).

Given that the PSS-Fa scores of unmarried patients were slightly higher than those of married patients, we conducted a Kruskal–Wallis test. The results showed a chi-square value (H) of 25.025 with 2 degrees of freedom, and both the asymptotic and exact significance levels were 0.000 (*p* < 0.05), indicating a significant difference in family support scores among different marital statuses. Further post hoc multiple comparisons using Dunn’s test revealed the following:

∙ There was a significant difference in family support scores between married individuals and those who were divorced or widowed (*p* < 0.001).

∙ There was also a significant difference in family support scores between unmarried individuals and those who were divorced or widowed (*p* = 0.002).

∙ There was no significant difference in family support scores between married and unmarried individuals (*p* = 1.000).

These findings suggest that while marital status does influence perceived family support, the distinction is most pronounced when comparing those who are married or unmarried with those who are divorced or widowed. Therefore, the lack of significant difference between married and unmarried individuals underscores the complexity of this relationship and highlights the need for further investigation into the factors contributing to perceived family support within these subgroups. Future research with a larger sample size may provide more definitive insights into the nuanced relationship between marital status and perceived family support.

### 3.2 Comparison of Self-Management Behaviors of Patients With Different Levels of Family Support

Table 2 presents the self-management behaviors of patients with different levels of family support. The overall mean score of self-management behaviors for the 76 patients was 140.80 ± 20.46 points. The mean scores and standard deviations for each self-management behavior item were as follows: medication (16.01 ± 2.00), nutrition (15.59 ± 2.06), exercise (13.77 ± 3.04), healthcare (42.07 ± 6.90), self-monitoring (22.10 ± 6.96), and seeking medical care (31.23 ± 6.14). The distribution of self-management behavior scores among patients with different levels of family support conformed to a normal distribution. Notably, patients with high levels of family support exhibited significantly higher scores in dietary behavior, self-monitoring behavior, and seeking medical care behavior compared to those with low levels of family support, with statistically significant differences (*p* < 0.05).

**Table 2.** **Comparison of self-management behavior scores between patients with high and low levels of family support**.

Group	n	Medication	Nutrition	Exercise	Health caring	Self-monitoring	Medical-seeking	Total points
High level	39	16.3 ± 2.1	16.2 ± 1.6	14.2 ± 2.7	43.3 ± 5.5	23.9 ± 6.9	32.7 ± 5.5	146.5 ± 18.3
Low level	37	15.8 ± 1.9	15.0 ± 2.3	13.3 ± 3.4	40.8 ± 8.0	20.2 ± 6.2	29.7 ± 6.5	134.8 ± 21.2
*t*		1.084	2.643	1.268	1.574	2.373	2.132	2.579
*p*-value		0.282	0.010	0.209	0.120	0.020	0.036	0.012

Note: Family support levels definitions: ∙ High level: patients scoring ≥11 on the PSS-Fa scale, indicating they receive substantial emotional, instrumental, informational, and appraisal support from their family members. ∙ Low level: patients scoring <11 on the PSS-Fa scale, indicating limited or insufficient support from their family members. Independent samples *t*-tests were used to compare the means of self-management behavior scores between the high and low family support groups.

**Fig. 1.**
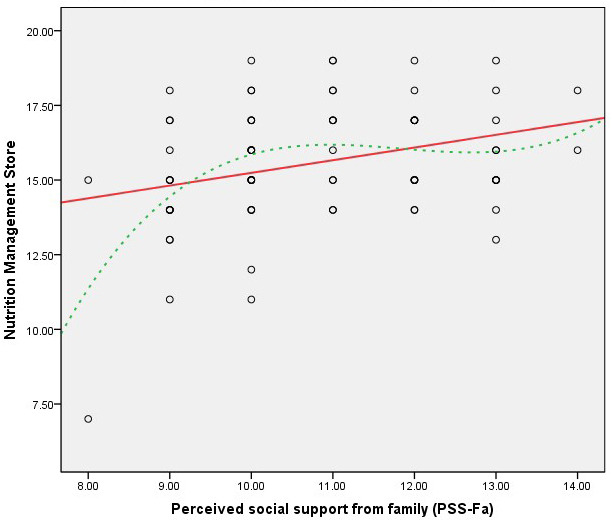
**Scatter plot of PSS-Fa vs. nutrition management (linear model *R_L_^2^* = 0.095; cubic polynomial model *R^2^* = 0.205)**. Note: the red solid line represents the linear fit, and the green dashed line depicts the cubic polynomial fit.

**Fig. 2.**
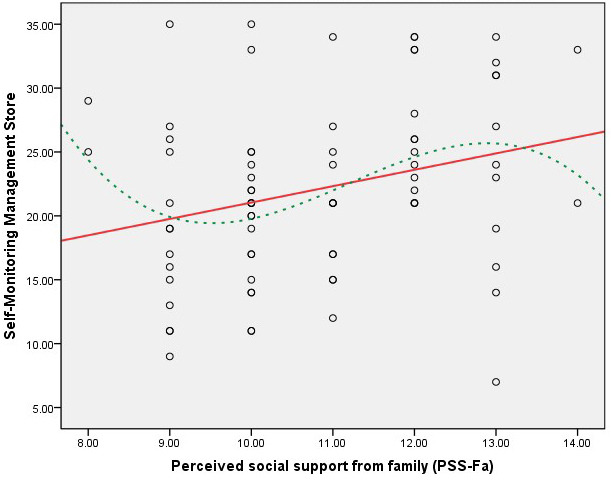
**Scatter plot of PSS-Fa vs. self-monitoring management (linear model *R_L_^2^* = 0.076; cubic polynomial model *R^2^* = 0.116)**. Note: the red solid line represents the linear fit, and the green dashed line depicts the cubic polynomial fit.

**Fig. 3.**
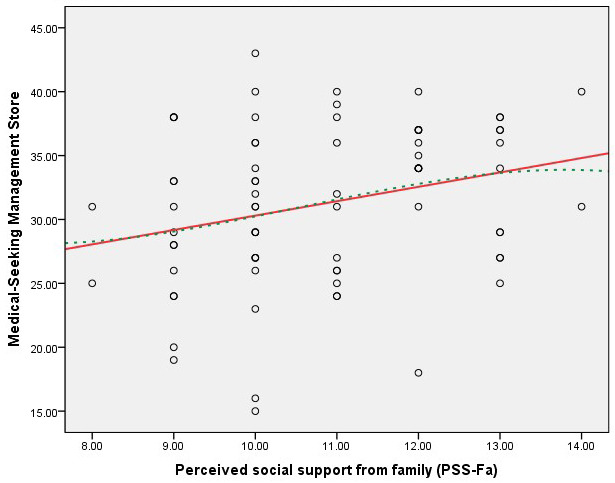
**Scatter plot of PSS-Fa vs. medical-seeking management (linear model *R_L_^2^* = 0.076; cubic polynomial model *R^2^* = 0.077)**. Note: the red solid line represents the linear fit, and the green dashed line depicts the cubic polynomial fit.

**Fig. 4.**
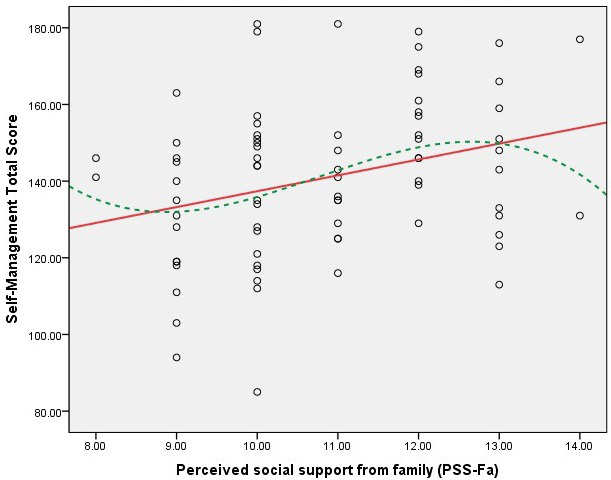
**Scatter plot of PSS-Fa vs. self-management total score (linear model *R_L_^2^* = 0.092; cubic polynomial model *R^2^* = 0.111)**. Note: the red solid line represents the linear fit, and the green dashed line depicts the cubic polynomial fit.

Figs. 1,2,3,4 present scatter plots comparing PSS-Fa scores with self-management behavior scores to confirm the linearity assumption for Pearson’s correlation analysis. These figures show the relationship between family support and specific self-management behaviors, along with fitted lines for both linear and cubic polynomial models. These findings indicate that higher levels of family support are associated with better self-management behaviors in these specific areas.

### 3.3 Correlation Analysis of Patients’ Family Support and Self-Management Behavior

Table 3 presents the results of the correlation analysis between PSS-Fa scores and self-management behavior scores one month after cardiac surgery. The study revealed a positive correlation between the PSS-Fa score and the total self-management behavior score (r = 0.303, *p* < 0.05).

**Table 3.** **Correlation analysis of patients’ family support and self-management behavior (n = 76)**.

Item	PSS-Fa	Medication	Nutrition	Exercise	Health caring	Self-monitoring	Medical-seeking	Self-management
PSS-Fa	1.000	0.085	0.308**	0.231*	0.157	0.276*	0.275*	0.303**
Medication	0.085	1.000	0.364**	0.500**	0.397**	0.597**	0.279*	0.630**
Nutrition	0.308**	0.364**	1.000	0.248*	0.143	0.368*	0.267*	0.428**
Exercise	0.231*	0.500**	0.248*	1.000	0.569**	0.706**	0.638**	0.847**
Health caring	0.157	0.397**	0.143	0.569**	1.000	0.567**	0.234*	0.739**
Self-monitoring	0.276*	0.597**	0.368*	0.706**	0.567**	1.000	0.547**	0.897**
Medical-seeking	0.275*	0.279*	0.267*	0.638**	0.234*	0.547**	1.000	0.715**
Self-management	0.303**	0.630**	0.428**	0.847**	0.739**	0.897**	0.715**	1.000

Note: * indicates significance *p* < 0.05, ** indicates significance *p* < 0.01.

Specifically, the correlation analysis showed statistically significant positive correlations for the following self-management behavior subdomains:

∙ Nutrition management: r = 0.308, *p* < 0.05

∙ Exercise management: r = 0.231, *p* < 0.05

∙ Self-monitoring management: r = 0.276, *p* < 0.05

∙ Medical-seeking management: r = 0.275, *p* < 0.05

However, no significant correlations were found between PSS-Fa scores and the following subdomains:

∙ Medication management: r = 0.085, *p* > 0.05

∙ Health care management: r = 0.157, *p* > 0.05

The lack of significant correlations between medication and healthcare management may be attributed to the nature of cardiac surgery. Patients undergoing cardiac surgery often have large surgical wounds and require prolonged treatment, which may enhance their self-management abilities in these specific areas. As a result, the influence of family support on these aspects of self-management may be less pronounced.

### 3.4 Independent Effects of Family Support on Self-Management Behaviors in Postoperative Cardiac Patients

To further identify the key factors influencing self-management behaviors in postoperative cardiac patients, we performed a one-way analysis of variance and multiple linear regression analyses. Univariate analysis revealed that patients’ total self-management behavior scores differed significantly with respect to age (*F* = 105.845), education level (*F* = 52.765), marital status (*F* = 24.367), monthly household income (*F* = 18.610), type of health insurance (*F* = 22.165), presence of comorbidities (*F* = 3.748), cardiac function (*F* = 5.648), and anxiety or depression status (*F* = 22.937), all *p* < 0.05. However, no significant differences were observed concerning gender (*t* = 0.236) and diagnosis (*F* = 1.566), all *p* > 0.05.

Multiple linear regression analysis: Multiple linear regression analysis was conducted to determine the significant factors influencing self-management behaviors. The results indicated that PSS-Fa score, age, and education level significantly predict self-management behaviors in postoperative cardiac patients. Specifically:

∙ PSS-Fa: *B* = 2.284, *SE* = 0.797, β’ = 0.167, *t* = 2.866, *p* = 0.000.

∙ Age: *B* = –18.918, *SE* = 2.948, β’ = –0.661, *t* = –6.417, *p* = 0.000.

∙ Education level: *B* = 9.396, *SE* = 3.423, β’ = 0.845, *t* = 5.992, *p* = 0.000.

The model’s overall fit statistics are as follows:

∙ *R^2^* = 0.821, indicating that the model can explain approximately 82.1% of the variance in self-management behaviors.

∙ *F* = 33.722: this is the F-statistic for the overall significance of the model, with a *p*-value less than 0.05 confirming its statistical significance.

Table 4 presents the results of multiple linear stepwise regression analyses of factors influencing self-management behavior in patients with different levels of family support.

**Table 4.** **Multiple linear regression analysis of factors influencing self-management behavior in different patients**.

Predictors	*B*	S*E*	β’	*t*	*p*-value	95% CI
PSS-Fa	2.284	0.797	0.167	2.866	0.000	[0.693, 3.874]
Age	–18.918	2.948	–0.661	–6.417	0.000	[–24.805, –13.032]
Education level	9.396	3.423	0.845	5.992	0.000	[3.439, 10.231]
Marital status	–2.880	0.478	–0.034	–0.602	0.550	[–1.243, 0.667]
Monthly income	3.166	2.920	0.093	1.084	0.282	[–2.664, 8.996]
Cardiac function	1.482	1.666	0.052	0.889	0.377	[–1.845, 4.809]
Complication	–1.811	1.229	–0.089	–1.473	0.145	[–4.264, 0.643]
Medical insurance type	–3.350	3.181	–0.092	–1.053	0.296	[–9.702, 3.001]
Anxiety/depression	–3.491	2.386	–0.113	–1.463	0.148	[–8.255, 1.272]

## 4. Discussion

The primary objective of CR is to induce long-term health behavioral changes and integrate regular physical activity and exercise training into daily life [22]. Our study provided important insights into the level of family support and self-management behaviors of patients after cardiac surgery one month postoperatively and identified relevant influencing factors. The results showed that the PSS-Fa score of patients after cardiac surgery one month postoperatively was 10.82 ± 1.50 points, which was moderate to high. Potential reasons for this result include that cardiac surgery patients have a longer recovery time and more complications and have higher requirements for postoperative CR. Hence, the degree of attention family members pay to the patient, and their disease is higher [23].

The potential factors influencing the level of family support of patients include that the PSS-Fa scores of women were higher than those of male patients, and the family support scores of married or unmarried patients were significantly higher than those of divorced or widowed patients, suggesting that spouses can provide more psychological support than other family members. Higher education was also associated with higher family support, which aligns with the results of patients with chronic heart failure [24]. Regarding the mechanism of gender differences in family support, we believe that women usually have better communication skills and can express their needs and obtain support more effectively. In the Chinese cultural context, women, as the central bond of the family, tend to take on the role of caregiver and thus are more likely to receive family support [25]. Furthermore, higher education was associated with higher family support, possibly because highly educated patients have better tolerance for major setbacks, are more willing to learn about the disease and prognosis, and actively communicate with their families for support [26]. In addition, the findings showed that married patients had significantly higher PSS-Fa scores than divorced or widowed patients, which may be because spouses can provide more psychological support to patients than children, parents, or other family members. Spouses provide more emotional comfort and help in real life, which is especially important for patients who lack channels of expression and emotional comfort [27]. However, it is noteworthy that unmarried patients showed a slightly higher mean PSS-Fa score compared to married patients. This suggests that other factors beyond marital status may contribute to perceptions of family support among unmarried individuals. Indeed, strong social networks outside of marriage, such as close friendships or extended family relationships, could play a significant role in this context. Unmarried individuals might rely on these alternative support systems to compensate for the absence of spousal support, leading to comparable or even higher levels of perceived family support [28]. Moreover, future research should explore the underlying mechanisms through which these factors influence family support perceptions.

Cardiac surgery patients require long-term CR to restore cardiac function, and better self-management ability is essential for ensuring that patients adhere to medical advice during the rehabilitation process. The results of this study showed that the total score of self-management behavior in postoperative cardiac surgery patients ranged from 85 to 181, with a mean of 140.80 ± 20.46. This indicates that their self-management behaviors were generally at an intermediate to upper level. As a percentage of the total score in descending order, the mean scores of each dimension in the self-management behavior scale were medication-taking, nutrition, health care, medical-seeking, exercise, and self-monitoring. Postoperative administration of anticoagulant drugs is a crucial component in cardiac surgical treatment, which requires patients to recognize associated complications and possess good self-management skills, avoid foods that interfere with drug metabolism, and seek medical care promptly if complications arise [29]. This study showed that patients with high-level and low-level family support demonstrated good compliance with their anticoagulant medications, recognized the importance of taking them, and could take them promptly and in the correct dosage. However, the patients’ self-monitoring management scores were relatively low, suggesting they could not correctly monitor postoperative complications. This may be due to a lack of knowledge and awareness of the importance of self-monitoring among patients and their family members [30]. Therefore, future discharge instructions should include information on disease self-monitoring to improve patients’ self-monitoring abilities. Additionally, the exercise management scores in this study were consistently low, which may be related to patients’ lack of cognitive understanding, insufficient social support, and fear of exercise [31].

As individuals within a social network, patients’ behavior is closely linked to their psychological state and social environment [32]. The results of this study identified several factors influencing the self-management behavior of postoperative cardiac patients, including age, education level, marital status, monthly family income, self-payment for medical care, comorbidity with other illnesses (such as hypertension), cardiac function class, and the presence of anxiety or depression. Multiple linear regression analysis showed that PSS-Fa, age, and education level were significant factors influencing self-management behaviors of postoperative cardiac patients, with PSS-Fa and education level showing a significant positive effect. In contrast, age showed a significant negative impact (*R^2^* = 0.821, *F* = 33.722, *p* < 0.05). As an extension of individual social support, family support is crucial in providing emotional and practical assistance. A study analyzing the health behaviors of older patients aged 70 and above in China found that a higher level of family social capital indicated that patients were more likely to receive support and behavioral supervision from family members, leading to active adjustments in their health-related behaviors [33]. China’s social security system is rapidly developing and improving compared to Western countries. Although several policies and measures have recently been introduced to support workers in balancing work and family responsibilities, shortcomings remain at the implementation level. This suggests that more attention is required to build a family-supportive environment suitable for the national context from the organizational culture perspective to improve the overall quality and efficiency of healthcare services [34]. Additionally, patients with higher education levels and comorbid conditions exhibited better self-management behaviors, which can be attributed to their higher cognitive levels and greater awareness of health-related issues. Higher education levels may enhance patients’ understanding of their condition and the importance of adhering to prescribed treatments. Moderate to severe anxiety or depression is common in patients undergoing cardiac surgery and can persist for extended periods, significantly impacting their self-management behavior after discharge [35]. In this study, 76.31% of patients experienced anxiety or depression, highlighting the importance of assessing and addressing patients’ psychological status. Thus, interventions such as music therapy, deep breathing, and progressive muscle relaxation should be incorporated into post-discharge follow-up to help manage these conditions [36].

Our results highlight the significant correlation between family support and self-management behaviors in cardiac surgery patients. Moreover, this study emphasizes the necessity of enhancing family support as an integral component of CR for patients. Furthermore, we underscore the importance of incorporating international comparisons in future studies to improve the identification of unique characteristics of Chinese patients and provide more practical clinical recommendations tailored to the local context. The health ecology model [37] posits that numerous factors, including health services, the social environment, physical life conditions, and individual attributes, are interdependent and mutually influential, collectively impacting individual health. Consequently, when formulating health promotion strategies for postoperative cardiac patients, it is imperative to consider the varying levels of family support and tailor HBCR programs.

## 5. Study Limitations

Despite the valuable insights provided by this study, several limitations should be acknowledged. First, this study chose patients discharged from our hospital after cardiac surgery treatment between January 2024 and June 2024. This specific time frame and single-center approach may introduce selection bias, limiting the generalizability of the findings to a broader population. Future studies should consider a multi-center design and a longer recruitment period to ensure a more diverse and representative sample. At the same time, the research team assessed the level of family support and self-management behavioral ability one month after discharge. This cross-sectional design does not capture the process or trend of changes in family support and self-management ability over time, limiting the study’s understanding of dynamic changes. A longitudinal study could track these variables over a longer period, providing a more in-depth understanding of their dynamics and potential causal relationships. In addition, this study analyzed the factors affecting self-management behavioral competence based on patients’ sociodemographic and clinical characteristics; however, it did not explore other potential factors such as socioeconomic status, access to healthcare resources, or cultural influences. Future research could incorporate a broader range of variables to provide a more comprehensive understanding of the factors influencing self-management behaviors. Lastly, with a sample size of 76 patients, the study may have limited statistical power to detect small to moderate effects. This limitation might result in type II errors (failing to detect true effects). Future studies should perform a priori power analysis to determine the minimum sample size required to achieve adequate statistical power for detecting meaningful effects. The failure of this study to directly measure CR participation in all patients is an important limitation. Although we highlighted significant correlations between family support and self-management behaviors, the lack of specific data on CR participation limits a comprehensive understanding of the overall recovery pathway. Future studies should include data collection on CR participation to more fully assess the relationship between family support, self-management behaviors, and CR participation.

This study has several strengths: The present study focused on HBCR to investigate the level of family support and self-management ability of patients and analyzed the factors influencing them, providing valuable insights for developing targeted interventions in the future. For example, patients with low levels of family support could be targeted with additional family support or psychological interventions.

## 6. Conclusions

CR is a critical component of care for patients with cardiovascular disease, yet its implementation remains nascent in China. Many hospitals currently lack the infrastructure and resources to provide comprehensive CR services. As an alternative strategy, HBCR holds promise for increasing patient participation rates. Our study demonstrated that higher levels of family support are positively associated with improved self-management abilities post-cardiac surgery, which in turn enhances the effectiveness of postoperative CR. Specifically:

∙ Gender: female patients exhibited significantly higher PSS-Fa scores than males, indicating a tendency to receive greater family support.

∙ Education level: individuals with higher educational attainment demonstrated significantly higher PSS-Fa scores, suggesting better access to family support and self-management practices.

∙ Marital status: Married individuals had the highest mean PSS-Fa scores, though no significant difference was found between married and unmarried individuals. Both groups reported significantly higher family support than those divorced or widowed.

The future management of patients following cardiac surgery should apply targeted intervention measures to address the diverse needs of different patient categories. These interventions could include:

(1) The development of tailored programs that empower both male and female patients to communicate their needs effectively and access appropriate support.

(2) The implementation of initiatives to enhance health literacy and coping skills among patients with varying educational backgrounds, promoting better family support and self-management.

(3) The provision of counseling services to strengthen spousal relationships and improve emotional support for married patients. For unmarried individuals, alternative support systems such as community groups, online forums, and peer support networks should be established to compensate for the absence of spousal support.

(4) We recommend targeted interventions to address the apparent deficiencies in patient self-monitoring and exercise management.

A detailed exercise program tailored to each post-cardiac surgery patient should be implemented based on existing exercise prescribing principles and practice guidelines; for example, it is recommended to perform moderate-intensity aerobic exercises, such as brisk walking or bicycling, at least three times a week; to continue for more than 30 minutes at a time, incorporating a 5–10-minute warm-up and cool-down period. To ensure the safety and effectiveness of exercise, the patient’s progress should be assessed regularly, and the exercise program should be adjusted accordingly. At the same time, patients are encouraged to record their daily activities so that their physicians can understand and evaluate their physical responses and instruct them accordingly [38].

Regarding self-monitoring, patients are taught how to correctly measure key indicators such as blood pressure and heart rate and explain the significance of these data. Smart devices or apps can also assist with monitoring, enabling patients to complete self-checks easily at home. Thus, by addressing these factors, healthcare providers can significantly improve family support and encourage patients to adopt healthier behaviors, ultimately leading to better clinical outcomes and enhanced quality of life.

## Data Availability

The datasets used and/or analyzed during the current study are available from the corresponding author upon reasonable request.

## Abbreviations

CR, cardiac rehabilitation; HBCR, home-based cardiac rehabilitation; PSS-Fa, perceived social support from family; AD, aortic dissection; CT, cardiac tumor; VHD, valvular heart disease; ASD, atrial septal defect; SD, standard deviation.
